# Dietary Calcium and Dairy Modulation of Oxidative Stress and Mortality in aP2-Agouti and Wild-type Mice

**DOI:** 10.3390/nu1010050

**Published:** 2009-08-14

**Authors:** Antje Bruckbauer, Michael B. Zemel

**Affiliations:** Department of Nutrition, The University of Tennessee, Knoxville, TN 37996-1920, USA; Email: abruckba@utk.edu

**Keywords:** aging, lifespan, oxidative stress, inflammatory stress, dietary calcium, dairy

## Abstract

Oxidative and inflammatory stress have been implicated as major contributors to the aging process. Dietary Ca reduced both factors in short-term interventions, while milk exerted a greater effect than supplemental Ca. In this work, we examined the effects of life-long supplemental and dairy calcium on lifespan and life-span related biomarkers in aP2-agouti transgenic (model of diet-induced obesity) and wild-type mice fed obesigenic diets until their death. These data demonstrate that dairy Ca exerts sustained effects resulting in attenuated adiposity, protection against age-related muscle loss and reduction of oxidative and inflammatory stress in both mouse strains. Although these effects did not alter maximum lifespan, they did suppress early mortality in wild-type mice, but not in aP2-agouti transgenic mice.

## 1. Introduction

Oxidative stress and inflammatory stress have been implicated as a cause of tissue damage in multiple organ systems leading to the development of chronic diseases such as obesity, diabetes, hypertony and atherosclerosis [1,2,3]. They are also recognized as major factors contributing to the physiological process of aging [4,5]. Dietary calcium appears to play a significant role in regulating reactive oxygen species (ROS) production and inflammation in adipocytes [6], and may therefore contribute to a reduction of metabolic disorders and a prolonged lifespan. The major intracellular source of ROS production and free radicals in mammalian cells are mitochondria [7]. Under normal physiological conditions, mitochondria generate ROS only at very low levels. However, several conditions, including obesity, hyperglycemia and hyperlipidemia can promote ROS production leading to oxidative damage to DNA, lipids and proteins. The resulting mitochondrial dysfunction can lead to increased ROS production with subsequent further damage and progressive decline of cellular and tissue functions due to energy depletion [7]. Supporting evidence shows a strong correlation between aging, increased mitochondrial ROS production and mitochondrial dysfunction [8,9,10,11]. In addition, inflammatory mediators such as tumor necrosis factor alpha (TNF-α) can induce oxidative stress by up-regulating NADPH oxidase and altering the mitochondrial redox state [12,13].

As a cellular defense against oxidative stress, mammalian cells have developed an array of antioxidant enzymes and proteins to dispose ROS under normal physiological conditions. These include superoxide dismutase (SOD), glutathione peroxidase (GPx) and catalase. However, the age associated decline of the enzyme activities coupled with an increase in intracellular ROS production can overwhelm the capacity of this defense system, resulting in increased oxidative stress [4,7,14].

Previous work from our laboratory has demonstrated an anti-obesity effect of calcium rich diets which we have proposed to be mediated, in part, by calcium suppression of circulating 1α,25-dihydroxyvitamin D (calcitriol) [15,16,17]. Calcitriol promotes glucocorticoid production in adipocytes, favoring fat storage in visceral rather than subcutaneous depots [18]. Visceral adiposity is characterized by a low-grade systemic inflammation and is associated with the development of metabolic disorders [19]. Calcitriol also regulates ROS production by adipocytes [6]. Consequently, we have shown that suppression of calcitriol by dietary calcium inhibits adipocyte-derived inflammatory cytokine expression [20] and reduces oxidative stress in murine and human adipocytes [6,21]. We also demonstrated that dairy products exert greater effects than supplemental calcium, and have proposed that this increased effect is due to additional components in dairy such as branched-chain amino acids (BCAA) and angiotensin converting enzyme inhibitor (ACEi) peptides [22,23]. 

Considering the protective role of dietary calcium and other dairy components against oxidative and inflammatory stress, which otherwise accelerate the process of aging, the present study was designed to evaluate the effects of dietary calcium from both non-dairy and dairy sources on mouse lifespan and on lifespan-related biomarkers. We utilized aP2-agouti transgenic mice because we have already demonstrated short-term effects of calcium on adiposity, oxidative stress and inflammatory stress in this model [6,20,23], and because these mice are susceptible to diet-induced obesity [24,30,31]. These mice express normal agouti protein under the control of the aP2 promoter in adipose tissue [24], thereby mimicking the human pattern of agouti expression. They are not obese on a standard AIN93 G diet but develop mild to moderate obesity (25–40% increase in adipose tissue mass) when fed high sucrose/high fat diets for six to 12 weeks [24,30]. In order to gain a more complete understanding of the effect of this genotype, this study was also done in wild type littermates.

## 2. Materials and Methods

### 2.1. Animals and Diets

Six to eight week old aP2-agouti mice of both genders (n = 197) and their littermate controls (n = 186) from our colony were randomly divided into three diet groups. Group 1 (control group) was fed a modified AIN93G diet with suboptimal calcium (calcium carbonate, 0.4%) and with sucrose as sole carbohydrate source, providing 64% of energy. Fat was increased to 25% of energy with lard. The sub-optimal level of calcium utilized for group 1 does not produce calcium deficiency, as it provides 80% of the recommended level (0.5%) in mice. Instead, this calcium level results in a compensatory endocrine response, characterized by elevations in plasma calcitriol levels in order to prevent calcium deficiency symptoms [23]. Group 2 (high calcium group) and group 3 (milk group, NFDM) received the same diet as group 1, however modified to contain 1.2% calcium in form of calcium carbonate or to contain 50% of protein replaced by non-fat dry milk with 1.2% calcium, respectively. Diet composition is shown in Table 1. 

**Table 1:** Diet composition of the control, high calcium and non-fat dry milk (NFDM) diet groups.

	Diet		
	Control (0.4%)	High Ca (1.2%)	NFDM (1.2%)
*Ingredient (gm)*			
Casein, 80 Mesh	160	160	0
DL-Methionine	3	3	0
Sucrose	637.9	638.3	429.7
Cellulose	50	50	50
Soybean oil	100	100	41.8
Lard	10	10	65
Mineral Mix S10022B	7	7	7
Calcium carbonate	10	30	17.4
Potassium phosphate, monobasic	8	8	8
Potassium Citrate, 1 H2O	1.6	1.6	1.6
Vitamin Mix V10037	10	10	10
Choline Bitartrate	2.5	2.5	2.5
*t*-Butylhydroqinone	0.014	0.014	0.014
Milk, Nonfat, Dry	0	0	400
Total	1000.014	1020.414	1033.014
*Macronutrients (g/kgdiet)*			
Protein	143.8	143.8	144.8
Carbohydrate	647.9	648.3	647.7
Fat	110.0	110.0	110.0
Fiber	50	50	50
*Macronutrients (gm %)*			
Protein	14.4	14.1	14.0
Carbohydrate	64.8	63.5	62.7
Fat	11.0	10.8	10.6
Fiber	5.0	4.9	4.8
*Macronutrients (kcal/kg diet)*			
Protein	575	575	579
Carbohydrate	2592	2593	2591
Fat	990	990	990
Total	4157	4158	4160
*Macronutrients (kcal %)*			
Protein	14	14	14
Carbohydrate	62	62	62
Fat	24	24	24
Total	100	100	100
Calcium, gm	4.03	12.03	12.03
Phosphorus, gm	3.1	3.1	5.7
Potassium, gm	3.61	3.61	10.81

There were similar numbers of both genders and genotypes in each group (31–35 animals/subgroup). Three to four animals of the same gender and diet group were kept together in polypropylene cages at a room temperature of 22 ± 2 °C and a regime of 12 h light/dark cycle until their death. The animals had free access to water and their experimental food throughout the experiment. All animals were checked daily for any signs of disease or death and moribund animals (as defined by the facility veterinarian) were humanely euthanized. Weight was measured to the nearest gram at the beginning of the experiment and then at the beginning of each month until the death of the animals. Small subgroups (four males and four females) of each diet group and breed (48 animals per age group) were terminated under CO_2_ anesthesia at 28, 52 and 78 weeks of age to determine biochemical markers in adipose tissue, liver, muscle and blood. Blood was collected by cardiac puncture. The excised tissues were immediately weighed and used for further studies, as described below. This study was approved from an ethical standpoint by the University of Tennessee Institutional Animal Care and Use Committee.

### 2.2. Measurement of Blood Chemistry Markers

Blood glucose was determined in whole blood by using a glucometer. Insulin and adiponectin plasma levels were assessed by using Mouse Insulin and Adiponectin ELISA Kits, respectively (Linco Research, St. Charles, MO, USA). The levels of TNFα, IGF-I, IL6 and IL15 in plasma were measured by using the specific Mouse TiterZyme ELISA Kits from Assay Designs Inc. (Ann Arbor, MI, USA). Plasma CRP levels were calculated by using the Mouse C-reactive protein ELISA Kit (Life Diagnostics, Inc., West Chester, PA, USA). MDA in plasma was assessed by using the TBARS Assay Kit (ZeptoMetrix Corporation, Buffalo, NY, USA). 

### 2.3. Measurement of Cortisol Release

Perirenal fat tissue was incubated in KRB buffer for two hours. Cortisol release in the medium was determined by using the Cortisol Enzyme Immunoassay Kit from Assay Designs, Inc (Ann Arbor, MI, USA).

### 2.4. Determination of Intracellular ROS Generation in White Adipose Tissue (Perirenal and Retro-peritoneal Fat Pads)

Adipose tissue (retroperitoneal and perirenal) digestion and adipocytes preparation was prepared as described in [Ca^2+^]_i_ measurement [6]. Adipose tissue was washed several times with Hank’s balanced salt solution, then minced into small pieces and digested with 0.8 mg/mL type I collagenase in a shaking water bath at 37°C for 30 min. Adipocytes were then filtered through sterile 500-μm nylon mesh and cultured in KRB buffer supplemented with 1% fetal bovine serum (FBS). Cells were cultured in suspension and maintained in a thin layer at the top of culture media for 2h for cell recovery. Intracellular ROS generation was assessed by using 6-carboxy-2’,7’-dichloro-dihydrofluorescein diacetate (H2-DCFDA) as described [25]. Cells were loaded with H2-DCFDA (2 μmol/L) for 30 minutes. After washing twice with KRB, Fluorescence (emission 520 nm) and protein content were measured. The intensity of fluorescence is expressed as arbitrary units per μg protein.

### 2.5. Measurement of Enzyme Activities in Liver

The *Superoxide Dismutase activity* (SOD) was measured by using an assay kit from Cayman Chemical Company (Ann Arbor, MI, USA). Tissue samples were prepared according to manufacture’s instructions. Two hundred µL of diluted radical detector (tetrazolium salt) was added to 10 µL of sample or standard. The reaction was started by adding 20 µL of diluted xanthine oxidase. After 20 min of incubation the absorbance was read at 450 nm using a plate reader. One unit of SOD is defined as the amount of enzyme needed to exhibit 50 % dismutation of the superoxide radical. All three types of SODs (Cu/Zn-, Mn-, and Fe-SOD) were measured by the assay.

The *Catalase activity* was measured as described [26]. Tissues were rinsed with phosphate buffered saline three times to remove any red blood cells, and then homogenized in 5–10 vol. of cold buffer (50 mM potassium phosphate, pH 7.0). 1% Triton X-100 was added to the stock homogenate (1:9) and then further diluted with phosphate buffer (1:200). The reaction was started by adding 1 mL H_2_O_2_ at 20°C to 2 ml homogenate. The decrease of absorbance at 240 nm against a blank (1 mL of phosphate buffer instead of substrate) was recorded for about 30s. 

The *Glutathione peroxidase* (GPx) activity was measured using an assay kit from Oxford Biomedical Research (Oxford, MI, USA). Liver tissue was prepared according to manufacture’s instructions. Three hundred and fifty µL of assay buffer, 350 µL of NADPH Reagent and 70 µL of sample were pipetted into a cuvette. The reaction was started by adding 350 µL of diluted *tert*-butyl hydroperoxide. The change in absorbance at 340 nm was recorded for three minutes.

The *NADPH oxidase* activity was measured using the modified Quantitative Photometric Assay as described [27]. Freshly excised tissue was homogenized in 5 vol. of homogenization buffer (Krebs-Ringer-Phosphate buffer containing 50 mM HEPES, 100 mM NaCl, 5 mM KCl, 1 mM MgCl_2_/H_2_O, 1 mM NaH_2_PO_4_, 1 mM CaCl_2_, and 2 mM glucose, pH 7.4). Fifty µL of sample was incubated with 50 µL of PBS and 50 µL of phorbol myristate acetate (PMA) (1.625 µmol/L; Sigma Diagnostics) to initiate the respiratory burst. After 10 min of mixing, 50 µL of nitroblue tetrazolium (NBT) (2.4 mmol/L; Sigma Diagnostics) was added to each individual well. The rate of NBT reduction was monitored at 490 nm for 30 min and optical density was recorded every 5 min by a photometer for microplates, starting immediately after the addition of NBT. The NADPH oxidase activity is expressed as the mean of absorbance (A) values in the 30-min period.

*AMPK activity* was measured as described in [28]. Freshly excised tissue was immediately homogenized in 2 vol. of homogenization buffer (50 mM Tris/HCL, 250 mM mannitol, 50 mM NaF, 5 mM sodium pyrophosphate, 1 mM EDTA, 1 mM EGTA, 1 mM DTT, 0.1 mM phenylmethylsulphonyl fluoride, 1 mM benzanidine, 1 μg/mL soybean trypsin inhibitor). Homogenate was centrifuged at 5,000 × g for 10 min and supernatant was aliquoted and stored at -80 °C until further use. Reaction mixtures were prepared containing 5 µL of 1 mM [γ-^32^P] ATP, 5 µL of 1 mM AMP in HEPES-Brij buffer, 5 µL of 1 mM SAMS peptide in HEPES-Brij buffer and 5 µL of HEPES-Brij buffer. The reaction was initiated by the addition of 5 µL AMPK sample. After 30 min of incubation at 30°C, 15 µL was removed and spotted onto a P81 paper square. After washing the paper squares twice with 1% (v/v) phosphoric acid and once with acetone, they were laid out on a paper towel and allowed to dry. Dried filters were counted after immersing in 5 mL of scintillation cocktail. The AMPK activity is expressed as nanomoles of phosphate incorporated into substrate peptide per minute.

### 2.6. Measurement of mRNA Expression

#### Total RNA extraction:

A total cellular RNA isolation kit (Ambion, Austin, TX, USA) was used to extract total RNA from abdominal (inguinal and retroperitoneal) fat (three animals/subgroup), liver and soleus muscle (four animals/subgroup) according to manufacturer’s instruction. The concentration, purity and quality of the isolated RNA were assessed by measuring the 260/280 ratio (1.7–2.1) and 260/230 ratio (close to 2.0) by using the ND-1000 Spectrophotometer (NanoDrop Technologies Inc., DE, USA).

#### Quantitative real time PCR:

Gene expression of SOD (1 to 3), glutathione peroxidase, catalase, NADPH oxidase, TNFα, IL6, IL15, UCP2, UCP3, adiponectin, 11β-HSD, NFкB, PPARα, PPARγ, PPARσ, AMPK, telomerase, Sirt1 and Sirt3 in retroperitoneal fat, soleus muscle and liver was quantitatively measured by using an ABI 7300 Real Time PCR System (Applied Biosystem, Branchburg, NJ, USA) with a TaqMan 1000 Core Reagent Kit (Applied Biosystem). The primers and probes sets were obtained from Applied Biosystems TaqMan® Gene Expression Assays primers and probe set collection according to manufacturer’s instruction. Pooled adipocyte total RNA was serial-diluted and used to establish a standard curve; total RNAs of unknown samples were also diluted in the same range. Reactions of quantitative RT-PCR for standards and unknown samples were performed according to the instructions of the ABI 7300 Real Time PCR System and TaqMan 1000 Core Reagent Kit (Applied Biosystem). Data for all transcripts were normalized to a housekeeping gene (18S) and are presented as a ratio of the transcript of interest to 18S. 

### 2.7. Statistical Analysis

Data were evaluated by Multivariate Analysis of Variance (MANOVA) for statistically significant main effects of gender, genotype, diet and age and possible interactions between groups. Statistically significantly different means in subgroups were separated by the least significant difference test (p ≤ 0.05) using ANOVA. Data points were identified as outliers graphically with box plots and removed when they were extreme (out of 3 IQ range). Survival analysis was done by using the Kaplan Meier survival analysis. All data analysis was done with SPSS software (SPSS inc., Chicago, IL, USA). Data are expressed as mean ± standard error (SE). 

## 3. Results

Because of the complex design of this experiment we used a MANOVA approach in the first step of analysis. This approach permits assessment of main effects of diets while holding the other factors (genotype, gender, age) constant as well as to identify interactions between factors. In cases where there are no such interactions, this provides a substantial increase in effective sample size. Accordingly, the following section presents the main effects of diet on the combined subgroups, and only presents other main effects and interactions when statistically significant.

### Body weight and composition

**Figure 1:**
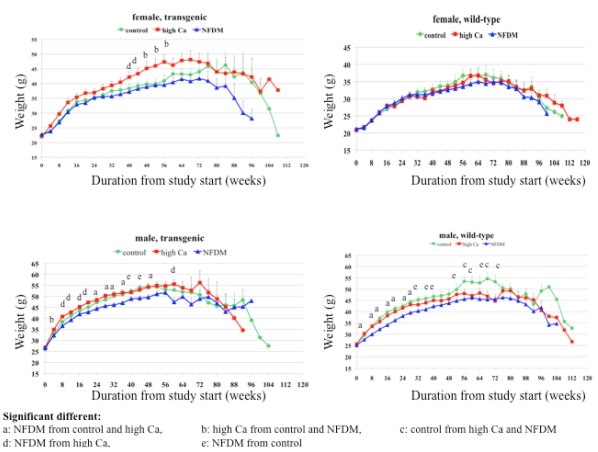
Mean weight distribution of all aP2-agouti transgenic and wild-type mice fed control, high calcium or NF DM diets over the study duration. Values are expressed as mean ± SE. Letters above curve denote significant differences between diet groups, p < 0.05.

Figure 1 shows the mean of body weight for each subgroup over the study duration. Table 2 and Table 3 list the percentage of total body fat (calculated from the sum of abdominal, perirenal, subscapular and epididymal (in male mice) fat pads divided by body weight) and the percentage of muscle tissue (calculated from the sum of soleus and gastrocnemius muscle divided by body weight), respectively. 

#### Males:

*Transgenic.* The milk diet group showed a lower weight compared to the high calcium group continuously over the first year and to the control group starting at month 6. Although, no difference in weight was found between high calcium and control group, there was a significant reduction in adipose tissue mass (Table 2). 

*Wild-type*. The weight in the milk diet was continuously lower compared to the control and high calcium diet group. This difference was associated with a reduction in adiposity (Table 2) and a prevention of muscle loss with age (Table 3) which was significantly different from the control group, but not the high calcium group. Although body weight in the high calcium group was not significantly different from the control group in the first year, it became statistically significant in the second year which was accompanied by a significant reduction in adiposity and a prevention of muscle loss compared to the control group at 78 weeks and 52 weeks of age, respectively.

**Table 2:** Percentage of total body fat calculated from inguinal, retroperitoneal, perirenal, subscapular and epididymal (in male) fat pads per body weight in aP2-agouti transgenic and wild-type mice fed control, high calcium or NFDM diets at 28, 52 and 78 weeks of age. Values are expressed as mean ± SE. Non-matching superscripts denote significant differences between diet groups, p < 0.05.

			Diet											
			control				high calcium				NFDM			
Gender	Genotype	Age	mean ± SE			n	mean ± SE			n	mean ± SE			n
female	wild	28 wk	6.53	±	2.89	4	12.10	±	1.57	4	9.24	±	2.22	4
		52 wk	18.53	±	2.18	4	14.37	±	1.07	4	12.16	±	3.07	4
		78 wk	19.42	±	1.49	3	17.84	±	3.73	4	12.98	±	1.44	3
		all	14.41	±	2.26	11	14.77	±	1.44	12	11.32	±	1.39	11
	transgenic	28 wk	19.27	±	1.69	4	16.90	±	4.72	3	12.99	±	0.65	4
		52 wk	19.24	±	4.39	4	22.13	±	1.15	4	16.52	±	1.20	4
		78 wk	23.07	±	2.94	5	23.84	±	4.61	3	18.62	±	3.33	4
		all	20.72	±	1.76	13	21.07	±	1.98	10	16.05	±	1.29	12
	both		17.83	±	1.53 ^a^	24	17.64	±	1.36 ^ab^	22	13.79	±	1.05 ^b^	23
male	wild	28 wk	12.43	±	0.87	4	12.73	±	0.66	4	10.08	±	0.75	4
		52 wk	15.33	±	1.84	4	15.06	±	0.19	4	13.65	±	0.36	4
		78 wk	17.47	±	1.96 ^a^	4	11.46	±	2.05 ^b^	4	13.44	±	1.11 ^ab^	4
		all	15.07	±	1.05 ^a^	12	13.08	±	0.79 ^ab^	12	12.39	±	0.64 ^b^	12
	transgenic	28 wk	12.54	±	1.11	4	12.38	±	0.49	5	12.87	±	0.94	4
		52 wk	16.67	±	1.25 ^a^	3	12.88	±	1.08 ^b^	4	13.18	±	0.51 ^b^	4
		78 wk	19.97	±	0.73	3	13.98	±	4.94	3	13.56	±	0.92	4
		all	16.00	±	1.23 ^a^	10	12.94	±	1.13 ^b^	12	13.20	±	0.43 ^ab^	12
	both		15.50	±	0.79 ^a^	22	13.02	±	0.68 ^ab^	24	12.79	±	0.39 ^b^	24
both	wild	28 wk	9.48	±	1.79	8	12.42	±	0.79	8	9.66	±	1.09	8
		52 wk	16.93	±	1.46 ^a^	8	14.72	±	0.52 ^ab^	8	12.91	±	1.46 ^b^	8
		78 wk	18.30	±	1.25 ^a^	7	14.62	±	2.30 ^ab^	8	13.24	±	0.84 ^b^	7
		all	14.76	±	1.18 ^a^	23	13.93	±	0.83 ^ab^	24	11.88	±	0.74 ^b^	23
	transgenic	28 wk	15.90	±	1.60	8	14.07	±	1.78	8	12.93	±	0.52	8
		52 wk	18.14	±	2.42	7	17.50	±	1.89	8	14.85	±	0.87	8
		78 wk	21.90	±	1.92	8	18.91	±	3.74	6	16.09	±	1.86	8
		all	18.67	±	1.21 ^a^	23	16.64	±	1.38 ^ab^	22	14.63	±	0.73 ^b^	24
	both	28 wk	12.69	±	1.42	16	13.24	±	0.96	16	11.29	±	0.72	16
		52 wk	17.49	±	1.32 ^a^	15	16.11	±	1.01 ^ab^	16	13.88	±	0.86 ^b^	16
		78 wk	20.22	±	1.24 ^a^	15	16.47	±	2.07 ^ab^	14	14.76	±	1.09 ^b^	15
		all	16.72	±	0.88 ^a^	46	15.23	±	0.81 ^ab^	46	13.28	±	0.55 ^b^	47

**Table 3:** Percentage of total muscle mass calculated from gastrocnemius and soleus muscle per body weight in aP2-agouti transgenic and wild-type mice fed control, high calcium or NFDM diets at 28, 52 and 78 weeks of age. Values are expressed as mean ± SE. Non-matching superscripts denote significant differences between diet groups, p < 0.05.

			Diet											
			control				high calcium				NFDM			
Gender	Genotype	Age	mean ± SE			n	mean ± SE			n	mean ± SE			n
female	wild	28 wk	0.33	±	0.05	4	0.40	±	0.04	4	0.32	±	0.04	4
		52 wk	0.25	±	0.04	4	0.28	±	0.02	4	0.41	±	0.09	4
		78 wk	0.35	±	0.06	3	0.32	±	0.05	4	0.36	±	0.03	3
		all	0.31	±	0.03	11	0.33	±	0.02	12	0.36	±	0.03	11
	transgenic	28 wk	0.28	±	0.02	4	0.35	±	0.10	3	0.38	±	0.05	4
		52 wk	0.22	±	0.02	4	0.24	±	0.04	4	0.30	±	0.04	4
		78 wk	0.18	±	0.02	5	0.27	±	0.08	3	0.33	±	0.08	4
		all	0.22	±	0.02 ^a^	13	0.28	±	0.04 ^ab^	10	0.34	±	0.33 ^b^	12
	both		0.26	±	0.02 ^a^	24	0.31	±	0.02 ^ab^	22	0.35	±	0.02 ^b^	23
male	wild	28 wk	0.29	±	0.05	4	0.28	±	0.05	4	0.43	±	0.06	4
		52 wk	0.13	±	0.01 ^a^	4	0.22	±	0.03 ^b^	4	0.25	±	0.03 ^b^	4
		78 wk	0.15	±	0.05 ^a^	4	0.19	±	0.04 ^a^	4	0.42	±	0.05 ^b^	4
		all	0.19	±	0.03 ^a^	12	0.23	±	0.02 ^ab^	12	0.36	±	0.04 ^b^	12
	transgenic	28 wk	0.23	±	0.05	4	0.26	±	0.02	5	0.23	±	0.01	4
		52 wk	0.15	±	0.02	3	0.16	±	0.03	4	0.24	±	0.03	4
		78 wk	0.19	±	0.005	3	0.29	±	0.04	3	0.23	±	0.08	4
		all	0.19	±	0.02	10	0.24	±	0.02	12	0.24	±	0.03	12
	both		0.19	±	0.02 ^a^	22	0.23	±	0.02 ^ab^	24	0.30	±	0.03 ^b^	24
both	wild	28 wk	0.31	±	0.03	8	0.34	±	0.04	8	0.38	±	0.04	8
		52 wk	0.19	±	0.03 ^a^	8	0.25	±	0.02 ^ab^	8	0.33	±	0.05 ^b^	8
		78 wk	0.24	±	0.05 ^a^	7	0.25	±	0.04 ^a^	8	0.39	±	0.03 ^b^	7
		all	0.25	±	0.024 ^a^	23	0.28	±	0.02 ^ab^	24	0.36	±	0.02 ^b^	23
	transgenic	28 wk	0.25	±	0.02	8	0.29	±	0.04	8	0.31	±	0.04	8
		52 wk	0.19	±	0.02 ^a^	7	0.20	±	0.03 ^ab^	8	0.27	±	0.03 ^b^	8
		78 wk	0.18	±	0.01	8	0.28	±	0.04	6	0.29	±	0.06	8
		all	0.20	±	0.01 ^a^	23	0.26	±	0.02 ^ab^	22	0.29	±	0.02 ^b^	24
	both	28 wk	0.28	±	0.02	16	0.32	±	0.03	16	0.34	±	0.03	16
		52 wk	0.19	±	0.02 ^a^	15	0.23	±	0.02 ^a^	16	0.30	±	0.03 ^b^	16
		78 wk	0.20	±	0.02 ^a^	15	0.27	±	0.03 ^ab^	14	0.34	±	0.04 ^b^	15
		all	0.23	±	0.01 ^a^	46	0.27	±	0.01 ^ab^	46	0.33	±	0.02 ^b^	47

#### Females:

*Transgenic.* The high calcium diet group exhibited the highest weight, and this difference reached statistical significance in older animals around 12 months of age. There was a trend for lower adiposity and higher percentage of muscle tissue in the milk diet group compared to the control diet group, however, it did not reach statistical significance.

*Wild-type*. No difference in weight, fat mass and muscle mass was found among the diet groups.

#### Summary:

There was a significant gender–diet interaction reflected in a significant weight difference in the male mice for both genotypes in the milk diet group compared to control and high calcium diet group but not in female mice. Although there was a comparable trend towards reduced adiposity in females, it only becomes significant when the groups are combined. In addition, the milk diet prevented the increase in fat mass and the loss in muscle mass with age compared to the control group when genotypes and genders were combined. Variation in weight evident in Figure 1 beginning at 24 months appear to be an artifact of the small number of surviving animals at that time point (1-3/group).

## 3.1. Oxidative stress

**Figure 2:**
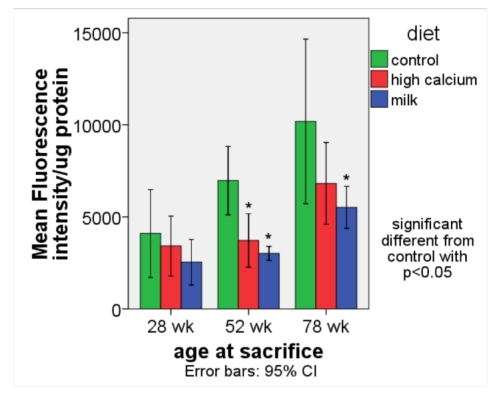
ROS production (expressed as fluorescence intensity/ μg protein) in white adipose tissue (retroperitoneal and perirenal fat) in aP2-agouti transgenic and wild-type mice (gender and genotype combined) fed control, high calcium or NFDM diets at 28, 52 and 78 weeks of age. Values are expressed as mean ± SE, (*n* = 4–5). * Significant different from control, p < 0.05.

*ROS production* (Figure 2). The age-related rise in ROS production was significantly blunted in the high calcium and milk diet groups compared to the control group.

*GPx gene expression in soleus* (Table 4). There were no diet interactions found; therefore data are collapsed for genotype, gender and age. The milk diet group had a significantly higher level of GPx gene expression than the control and high calcium diet groups.

**Table 4:** Glutathione Peroxidase (GPx) (soleus muscle) and Superoxide Dismutase 3 (SOD3) gene expression (soleus muscle and liver) and liver SOD enzyme activity of aP2-agouti transgenic and wild-type mice fed control, high calcium or NFDM diets (all three age groups combined). Gene expression data are normalized to 18S expression, enzyme activity to protein content. Values are expressed as mean ± SE. Non-matching superscripts denote significant differences between diet groups, p < 0.05.

			Diet											
			control				high calcium				NFDM			
Gene expression	Gender	Genotype	mean ± SE			n	mean ± SE			n	mean ± SE			n
GPx soleus	female	wild	0.77	±	0.06 ^a^	12	0.93	±	0.90 ^ab^	12	1.06	±	0.09 ^b^	12
		transgenic	0.81	±	0.08 ^a^	12	0.84	±	0.08 ^a^	11	1.20	±	0.14 ^b^	12
		both	0.79	±	0.05 ^a^	24	0.89	±	0.06 ^a^	23	1.14	±	0.08 ^b^	24
	male	wild	0.83	±	0.07	11	0.74	±	0.07	12	1.22	±	0.20	12
		transgenic	0.83	±	0.14	11	0.81	±	0.10	13	0.82	±	0.11	13
		both	0.83	±	0.07	22	0.77	±	0.06	25	1.01	±	0.12	25
	both	wild	0.80	±	0.05 ^a^	23	0.83	±	0.06 ^a^	24	1.14	±	0.10 ^b^	24
	both	transgenic	0.82	±	0.08	23	0.83	±	0.07	24	1.02	±	0.10	25
	both	both	0.81	±	0.04 ^a^	46	0.83	±	0.05 ^a^	48	1.07	±	0.07 ^b^	49
SOD 3 soleus	female	wild	0.73	±	0.09 ^a^	12	0.88	±	0.12 ^a^	12	1.31	±	0.19 ^b^	12
		transgenic	0.81	±	0.10	12	0.84	±	0.13	11	0.88	±	0.10	12
		both	0.77	±	0.07 ^a^	24	0.86	±	0.09 ^ab^	23	1.09	±	0.12 ^b^	24
	male	wild	0.96	±	0.08	11	0.90	±	0.17	12	1.27	±	0.26	12
		transgenic	0.80	±	0.07	11	0.89	±	0.09	13	0.85	±	0.14	13
		both	0.88	±	0.06	22	0.90	±	0.10	25	1.06	±	0.15	25
	both	wild	0.84	±	0.07 ^a^	23	0.89	±	0.11 ^ab^	24	1.30	±	0.16 ^b^	24
	both	transgenic	0.81	±	0.06	23	0.87	±	0.08	24	0.87	±	0.09	25
	both	both	0.82	±	0.05	46	0.88	±	0.07	48	1.07	±	0.09	49
SOD 3 liver	female	wild	0.48	±	0.07	12	0.79	±	0.22	12	0.86	±	0.27	12
		transgenic	0.62	±	0.11	12	0.52	±	0.13	11	0.78	±	0.26	12
		both	0.56	±	0.07	24	0.66	±	0.13	23	0.83	±	0.19	24
	male	wild	0.48	±	0.10	11	0.50	±	0.12	12	0.97	±	0.52	11
		transgenic	0.52	±	0.13	11	0.38	±	0.06	12	0.41	±	0.08	13
		both	0.51	±	0.08	22	0.44	±	0.07	24	0.67	±	0.25	24
	both	wild	0.49	±	0.06	23	0.65	±	0.13	24	0.92	±	0.28	23
	both	transgenic	0.58	±	0.09	23	0.45	±	0.07	23	0.59	±	0.14	25
	both	both	0.53	±	0.05	46	0.55	±	0.08	47	0.75	±	0.15	48
Enzyme activity														
SOD liver	female	wild	0.59	±	0.07 ^ab^	12	0.35	±	0.08 ^a^	12	0.83	±	0.17 ^b^	12
		transgenic	0.55	±	0.16	12	0.65	±	0.14	11	0.63	±	0.12	12
		both	0.57	±	0.90	24	0.49	±	0.08	23	0.73	±	0.10	24
	male	wild	0.54	±	0.16	11	0.48	±	0.10	12	0.76	±	0.18	11
		transgenic	0.34	±	0.08	11	0.37	±	0.07	12	0.59	±	0.14	13
		both	0.44	±	0.09	22	0.42	±	0.06	24	0.68	±	0.11	24
	both	wild	0.56	±	0.09 ^ab^	23	0.42	±	0.06 ^a^	24	0.79	±	0.12 ^b^	23
	both	transgenic	0.45	±	0.09	23	0.50	±	0.08	23	0.61	±	0.09	25
	both	both	0.50	±	0.06 ^a^	46	0.46	±	0.05 ^a^	47	0.70	±	0.07 ^b^	48

*SOD3 gene expression in soleus* (Table 4). There was a significant diet-genotype interaction. Wild type mice showed increased levels of SOD 3 expression in the milk diet group while no diet effect was found in the transgenic mice.

*SOD3 gene expression and enzyme activity in liver* (Table 4). There were no diet interactions found; therefore only main effects of diet are reported. There was an overall increase in SOD enzyme activity in liver in the milk diet group compared to high calcium and control group; however, this was not associated with a significant difference in gene expression. 

### Summary:

The age-related rise in ROS production was significantly attenuated by calcium rich diets. This was associated in the milk diet group with an increase in GPx and SOD3 gene expression in soleus muscle as well as an increase in liver SOD enzyme activity. We also examined other markers such as Catalase and NADPH oxidase gene expression and liver activity, AMPK liver activity, and plasma MDA levels; however, no significant differences among the diet groups were found (data not shown).

## 3.2. Inflammatory Stress

There was a statistically significant diet-gender interaction for adipocytokines (Table 5). Male mice showed lower levels of TNFα and IL6 gene expression in abdominal fat tissue in the milk diet, consistent with previous data from our laboratory. Levels in females were quite low compared to males and were unaffected by diet. Cytokines and other markers in plasma were unchanged (data not shown).

**Table 5:** Gene expression of IL6, IL15, TNFα and NFкB in inguinal fat tissue in aP2-agouti transgenic and wild-type mice fed control, high calcium or NFDM diets (all three diet groups combined). Data are normalized to 18S expression. Values are expressed as mean ± SE. Non-matching superscripts denote significant differences between diet groups, p < 0.05.

			Diet											
			control				high calcium				NFDM			
Gene expression	Gender	Genotype	mean ± SE			n	mean ± SE			n	mean ± SE			n
IL 6	female	wild	0.21	±	0.04	8	0.40	±	0.13	8	0.42	±	0.16	9
		transgenic	0.38	±	0.08	11	0.38	±	0.08	9	0.26	±	0.32	8
		both	0.31	±	0.05	19	0.39	±	0.07	17	0.35	±	0.08	17
	male	wild	1.45	±	0.86	8	2.18	±	0.67	10	0.57	±	0.22	8
		transgenic	6.23	±	2.12	8	2.87	±	1.15	8	0.51	±	0.07	9
		both	3.84	±	1.27 ^a^	16	2.49	±	0.62 ^a^	18	0.53	±	0.48 ^b^	17
	both	wild	0.83	±	0.44	16	1.39	±	0.43	18	0.49	±	0.13	17
	both	transgenic	2.85	±	1.09	19	1.56	±	0.61	17	0.39	±	0.05	17
	both	both	1.93	±	0.64	35	1.47	±	0.36	35	0.44	±	0.07	34
TNF α	female	wild	0.33	±	0.06	8	0.69	±	0.30	8	0.24	±	0.04	9
		transgenic	0.47	±	0.08	11	0.68	±	0.16	9	0.50	±	0.16	9
		both	0.41	±	0.05	19	0.69	±	0.16	17	0.37	±	0.08	18
	male	wild	1.49	±	0.47	8	2.39	±	0.39	10	0.73	±	0.13	9
		transgenic	3.83	±	0.74 ^a^	6	2.53	±	0.64 ^ab^	9	1.17	±	0.20 ^b^	9
		both	2.49	±	0.51 ^a^	14	2.46	±	0.35 ^a^	19	0.95	±	0.13 ^b^	18
	both	wild	0.91	±	0.27 ^ab^	16	1.64	±	0.32 ^a^	18	0.49	±	0.09 ^b^	18
	both	transgenic	1.65	±	0.47	17	1.60	±	0.39	18	0.84	±	0.15	18
	both	both	1.29	±	0.28	33	1.62	±	0.25	36	0.66	±	0.09	36
IL 15	female	wild	0.43	±	0.09	8	0.64	±	0.14	8	0.69	±	0.18	9
		transgenic	0.84	±	0.19	11	0.88	±	0.26	9	0.93	±	0.27	9
		both	0.66	±	0.13	19	0.77	±	0.15	17	0.81	±	0.16	18
	male	wild	0.85	±	0.22 ^a^	7	1.45	±	0.22 ^b^	10	0.61	±	0.09 ^a^	9
		transgenic	1.18	±	0.26	8	1.26	±	0.31	9	1.07	±	0.21	9
		both	1.02	±	0.18	15	1.36	±	0.18	19	0.84	±	0.12	18
	total	wild	0.62	±	0.12	15	1.09	±	0.16	18	0.65	±	0.10	18
	total	transgenic	0.98	±	0.16	19	1.07	±	0.20	18	1.00	±	0.17	18
	total	total	0.82	±	0.11	34	1.08	±	0.13	36	0.83	±	0.10	36
NFкB	female	wild	0.84	±	0.25	8	0.85	±	0.20	8	0.72	±	0.17	9
		transgenic	0.86	±	0.17	11	0.96	±	0.21	9	1.03	±	0.23	9
		total	0.85	±	0.14	19	0.91	±	0.14	17	0.88	±	0.15	18
	male	wild	1.27	±	0.38	8	1.58	±	0.22	10	0.83	±	0.16	9
		transgenic	1.49	±	0.47	7	1.09	±	0.26	9	1.17	±	0.22	9
		total	1.38	±	0.29	15	1.35	±	0.17	19	1.01	±	0.14	18
	both	wild	1.05	±	0.22	16	1.26	±	0.17	18	0.77	±	0.12	18
	both	transgenic	1.13	±	0.23	18	1.03	±	0.17	18	1.11	±	0.16	18
	both	both	1.09	±	0.16	34	1.14	±	0.12	36	0.94	±	0.10	36

## 3.3. Survival

**Figure 3:**
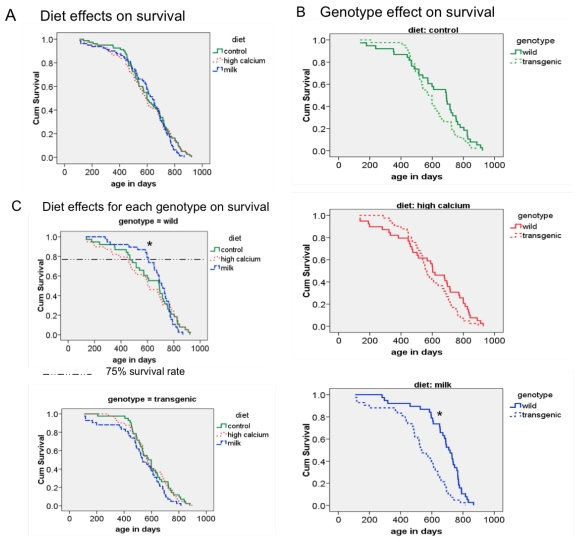
Survival curves of all aP2- agouti transgenic and wild-type mice fed control, high calcium or NFDM diets. A: Diet effect on survival independent of gender and genotype. B: Genotype effect on survival independent of diet. C: Diet effect for each genotype on survival. * Significant different, p < 0.05.

The survival analysis showed a longer lifespan in wild-type compared to transgenic mice for the milk diet group (Figure 3B). There was no overall diet effect on maximal lifespan (Figure 3A), although the milk diet significantly reduced the early mortality rate in wild-type mice as demonstrated by a significant increase in 75% survival rate (Figure 3C).

## 3.4. Additional Findings

Interestingly, we found a reduction of Sirt1 gene expression in abdominal fat (at 78 weeks) and in soleus muscle (Table 6). Also liver PPARα gene expression was reduced in the milk diet compared to the control group (Table 6).

**Table 6:** Sirt1 (inguinal fat and soleus muscle) and PPARα (liver) gene expression in aP2-agouti transgenic and wild-type mice fed a control, high calcium or NFDM diet. Data are normalized to 18S expression. Values are expressed as mean ± SE. Non-matching superscripts denote significant differences between diet groups, p < 0.05.

				Diet											
				control				high calcium				NFDM			
Gene expression	Gender	Genotype	Age	mean ± SE			n	mean ± SE			n	mean ± SE			n
Sirt1 abdominal fat	female	wild	all	0.88	±	0.28	8	0.97	±	0.17	8	0.82	±	0.16	8
		transgenic	all	0.12	±	0.22	11	1.28	±	0.25	9	0.91	±	0.21	9
		both	all	1.02	±	0.17	19	1.13	±	0.15	17	0.86	±	0.13	17
	male	wild	all	1.17	±	0.38 ^a^	8	1.43	±	0.16 ^b^	10	0.77	±	0.12 ^a^	9
		transgenic	all	1.28	±	0.24	8	1.02	±	0.25	9	1.10	±	0.22	9
		both	all	1.23	±	0.22	16	1.24	±	0.15	19	0.94	±	0.13	18
	both	wild	all	1.03	±	0.23 ^ab^	16	1.23	±	0.13 ^b^	18	0.79	±	0.09 ^a^	17
		transgenic	all	1.19	±	0.16	19	1.15	±	0.17	18	1.00	±	0.15	18
		both	28 wk	0.53	±	0.10	12	0.66	±	0.13	12	0.69	±	0.16	11
			52 wk	0.87	±	0.12 ^ab^	12	1.09	±	0.12 ^b^	12	0.56	±	0.08 ^a^	12
			78 wk	2.02	±	0.19 ^a^	11	1.81	±	0.11 ^a^	12	1.43	±	0.08 ^b^	12
	both	both	all	1.12	±	0.13	35	1.18	±	0.11	36	0.90	±	0.09	35
Sirt1 soleus	female	wild	all	0.86	±	0.12	11	1.02	±	0.11	11	0.78	±	0.08	12
		transgenic	all	1.07	±	0.08 ^a^	11	0.86	±	0.08 ^ab^	11	0.82	±	0.08 ^b^	12
		both	all	0.97	±	0.08	22	0.94	±	0.07	22	0.80	±	0.06	24
	male	wild	all	0.96	±	0.07	10	1.13	±	0.10	11	0.98	±	0.13	11
		transgenic	all	0.98	±	0.11	11	1.01	±	0.10	11	0.78	±	0.08	12
		both	all	0.97	±	0.04 ^ab^	21	1.07	±	0.07 ^b^	22	0.88	±	0.08 ^a^	23
	both	wild	all	0.91	±	0.07	22	1.07	±	0.07	22	0.88	±	0.08	23
		transgenic	all	1.03	±	0.05 ^a^	21	0.94	±	0.06 ^ab^	22	0.80	±	0.05 ^b^	24
		both	28 wk	0.79	±	0.05	14	0.76	±	0.05	15	0.69	±	0.06	15
			52 wk	1.04	±	0.09 ^ab^	15	1.20	±	0.10 ^b^	13	0.88	±	0.07 ^a^	16
			78 wk	1.07	±	0.06	14	1.07	±	0.07	16	0.93	±	0.10	16
	both	both	all	0.97	±	0.04 ^a^	43	1.00	±	0.05 ^a^	44	0.84	±	0.05 ^b^	47
PPAR α liver	female	wild	all	0.62	±	0.11	12	0.65	±	0.13	12	0.70	±	0.10	12
		transgenic	all	0.83	±	0.14 ^a^	12	0.79	±	0.14 ^a^	11	0.42	±	0.07 ^b^	12
		both	all	0.73	±	0.09	24	0.72	±	0.10	23	0.56	±	0.07	24
	male	wild	all	0.86	±	0.18	11	0.40	±	0.08	11	0.41	±	0.08	11
		transgenic	all	0.86	±	0.18	11	0.57	±	0.11	12	0.56	±	0.07	13
		both	all	0.86	±	0.12 ^a^	22	0.49	±	0.07 ^b^	23	0.49	±	0.05 ^b^	24
	both	wild	all	0.74	±	0.10	23	0.53	±	0.08	23	0.56	±	0.07	23
		transgenic	all	0.84	±	0.11 ^a^	23	0.68	±	0.09 ^ab^	23	0.49	±	0.05 ^b^	25
		both	28 wk	0.88	±	0.10 ^a^	15	0.57	±	0.09 ^b^	16	0.56	±	0.08 ^b^	17
			52 wk	0.47	±	0.06	16	0.55	±	0.11	15	0.45	±	0.07	15
			78 wk	1.04	±	0.17 ^a^	15	0.70	±	0.12 ^ab^	15	0.56	±	0.07 ^b^	16
	both	both	both	0.79	±	0.08 ^a^	46	0.61	±	0.06 ^ab^	46	0.53	±	0.04 ^b^	48

## 4. Discussion

Our data demonstrate that age-related changes in body composition were blunted by calcium-rich diets. This is consistent with our previous short-term findings, which show that calcium-rich diets contribute to a reduction in body fat mass in both humans and mice under eucaloric and hypocaloric conditions [15,29,30,31]. We have also noted that dairy foods exert significantly greater effects than supplemental sources of calcium, and have attributed this observation to other bioactive components in dairy products, such as branched-chain amino acids (BCAA) and angiotensin-converting enzyme inhibitor (ACEi) peptides [32]. Leucine is well recognized to stimulate muscle protein synthesis, and leucine supplementation decreases age-related loss in muscle mass in both rodents and humans [33,34,35]. Our previous data also indicate that leucine alters energy partitioning between adipose tissue and muscle by augmenting lipolysis in adipose tissue and stimulating fatty acid oxidation in muscle to supply the energy demand of the anabolic process of protein synthesis [22]. Consistent with these observations, data from this study show a greater effect of the milk diet than of the high calcium diet in preventing the age-related increase in adiposity and the age-related loss of muscle mass. 

The free radical theory of aging, originally proposed by Harman [36], postulates that the aging process is caused by accumulation of free radicals over time, resulting in oxidative damage and progressive deterioration of biological systems. Since mitochondria are the major production site of free radicals, this theory was extended to the mitochondrial theory of aging [37], which proposes that cumulative oxidative damage to mtDNA leads to a decline in bioenergetic function of mitochondria with a progressive decline of cellular and tissue functions. Other major sources of oxidative stress are ROS generating enzymes such as calcium-dependent NADPH oxidase which plays a crucial role in developing vascular diseases [38]. Furthermore, there is bidirectional interaction between intracellular calcium signaling and ROS with calcium activating ROS production by cellular enzymes and mitochondria, and ROS increasing intracellular calcium levels by activating calcium channels on plasma membrane and endoplasmatic reticulum [39,40]. Accordingly, our previous data demonstrate that decreasing intracellular [Ca^2+^] by dietary calcium can diminish ROS production, as increasing dietary calcium suppresses circulating calcitriol, which otherwise increases intracellular [Ca^2+^]_i_ in multiple cell types [16,17]. 

We have shown that suppression of calcitriol results in an inhibition of cellular NADPH oxidase gene expression [6], and in an increase in UCP production which diminishes mitochondrial membrane potential, thereby resulting in decreased ROS production [41]. In addition, dairy products further enhances this anti-oxidant effect [23], most likely mediated by the inhibition of the local adipocyte renin-angiotensin-system (RAS) by ACEi peptides in dairy products [42,43,44]. Data from this long-term study also demonstrate that dietary calcium significantly reduced the age-related rise in ROS, and that dairy exerted a greater effect than calcium carbonate. However, in contrast with our previous data, this reduction was not found in younger mice (28 weeks old) and was not associated with alterations of NADPH oxidase and UCP expression. Instead, we found an increase in antioxidant enzyme activity and associated gene expression in the milk diet. Since aging is associated with a decline in anti-oxidant enzyme activity [14,45], our observation that dairy components appear to attenuate this decline is likely to play a role in reducing ROS formation with aging. 

Oxidative stress can cause a dysregulation of adipocytokines [2,46], and data from this study indicate that this effect is attenuated by high calcium diets, as demonstrated by decreases in adipose tissue TNFα and IL6 expression. Consistent with this, we previously demonstrated that calcitriol can stimulate inflammatory cytokine expression in human and murine adipocytes [20] and that suppression of calcitriol by high calcium diets attenuates the inflammatory response in shorter-term studies [23]. An additional reduction is seen with dairy products; this is most likely mediated by ACEi peptides [47]. 

There was a significant gender-diet interaction for body weight and abdominal adipocytokine production. The milk diet significantly reduced body weight gain over the first year and reduced expression of TNFα and IL6 in visceral abdominal fat in male mice, while these effects were not found in females. Multiple studies have shown that sex hormones are responsible for gender specific differences in fat distribution and adipose metabolism [48,49]. Premenopausal females tend to accumulate fat in non-visceral areas such as hip and thighs while men develop visceral adiposity with a higher risk for metabolic disorders, as visceral fat is the major production site of adipocytokines [50,51,52]. Moreover, estrogens can cause an anti-inflammatory effect with inhibition of TNFα and IL6 in macrophages [53,54]. We have demonstrated that the response of visceral fat to dietary calcium is higher than that of subcutaneous fat [20]. In support of these concepts, the male mice showed markedly higher levels of adipocytokines compared to females. 

Since oxidative and inflammatory stress is a major contributor to aging, we hypothesized that a reduction of both by dietary calcium could influence the lifespan in mice. In support of this hypothesis, studies in short-lived animals such as nematodes and flies have shown that a reduction in oxidative stress increased the lifespan [55,56,57]. In addition, the senescence accelerated mouse (SAM) model is associated with many characteristic features of mammalian aging, increased oxidative stress and a shortened lifespan [58], and administration of a free radical scavenger prolonged the lifespan in this model [59]. On the other hand, modifying the antioxidant defense system resulted in contradicting findings in some models of aging. While over-expression of catalase in mice extended the life span [60,61], a reduction in SOD activity increased oxidative stress and cancer incidence but did not accelerate aging in mice [62]. Although the age-related increase of oxidative stress was reduced by the milk diet in our animals, we did not find any overall diet effect on mean and maximal survival; however, the milk diet decreased the early mortality in wild-type mice, as demonstrated by a significant increase in 75% survival rate. At this point, it is not clear why this effect is seen only in the wild-type mice. Since the agouti transgene is only locally expressed in adipose tissue, no systemic alterations were expected. However, the survival analysis revealed a shorter survival rate of the transgenic mice compared to the wild-type mice in the milk diet group. Therefore, it is possible that this overall genotype effect could have overwhelmed any possible diet interaction.

Interestingly, we found a decrease of sirtuin 1 (Sirt1) gene expression in abdominal adipose tissue and soleus muscle as well as a reduction of peroxisome proliferation activating receptor (PPAR) α gene expression in liver in the milk diet group. The sirtuins belong to a conserved family of deacetylases and have been linked to prevention of mitochondrial dysfunction, aging and metabolic disease [63,64]. In addition, the NAD+-dependent histone deacetylase Sirt1 regulates energy metabolism such as glucose homeostasis and fat metabolism [65]. PPARα, a ligand-activated transcription factor and mainly expressed in muscle and liver, is also involved in lipid metabolism and inflammation [66]. At this point, it is not clear how to interpret these findings, especially in the absence of data from other members of the sirtuin family. We speculate that the overall reduction in adiposity, oxidative and inflammatory stress by the milk diet acts as a negative feedback loop and counteracts Sirt1 and PPARα up-regulation.

In conclusion, data from the present study demonstrate that life-long feeding of a milk diet attenuates adiposity under eucaloric conditions and protects against age-related muscle loss. Consistent with previous findings, we show that dietary calcium reduces oxidative and inflammatory stress. Although these effects did not influence the maximum lifespan, they did suppress early mortality.
